# Health of Croydon

**Published:** 1857-01

**Authors:** 


### HEALTH OF CROYDON.

THE Board of Health, having addressed some inquiries to the Medical Officer of Health for Croydon, respecting the mortality and health of the inhabitants of that district, received an answer from Mr. Bottomley, to whom the letter went by mistake, as there is no medical officer of health in Croydon. Mr. Bottomley gave a very dismal history of the sanitary state of his district. Hereupon the Local Board of Health met, repudiated Mr. Bottomley's statements, declared that he had no authority to answer the letter, and drew up a report from the evidence of various medical men, ministers, and residents of the locality, proving that the town is in a healthy state, and that the new drainage system works exceedingly well. It is fair to say, that these inquiries on the part of the Local Board seem to have been conducted with impartiality and justice, and that Croydon is really improved in its sanitary resources.

